# Inflammasome and pyroptosis in autoimmune liver diseases

**DOI:** 10.3389/fimmu.2023.1150879

**Published:** 2023-03-08

**Authors:** Jixuan Wang, Zhiwen Sun, Jingri Xie, Wanli Ji, Yang Cui, Zongxiong Ai, Guoying Liang

**Affiliations:** ^1^ School of First Clinical Medicine, Heilongjiang University of Chinese Medicine, Harbin, China; ^2^ Department of Liver, Spleen and Stomach Diseases, First Affiliated Hospital of Heilongjiang University of Chinese Medicine, Harbin, China

**Keywords:** inflammasomes, pyroptosis, autoimmune hepatitis (AIH), primary biliary cholangitis (PBC), primary sclerosing cholangitis (PSC), gut microbiota

## Abstract

Autoimmune hepatitis (AIH), primary biliary cholangitis (PBC), primary sclerosing cholangitis (PSC), and IgG4-related sclerosing cholangitis (IgG4-SC) are the four main forms of autoimmune liver diseases (AILDs), which are all defined by an aberrant immune system attack on the liver. Most previous studies have shown that apoptosis and necrosis are the two major modes of hepatocyte death in AILDs. Recent studies have reported that inflammasome-mediated pyroptosis is critical for the inflammatory response and severity of liver injury in AILDs. This review summarizes our present understanding of inflammasome activation and function, as well as the connections among inflammasomes, pyroptosis, and AILDs, thus highlighting the shared features across the four disease models and gaps in our knowledge. In addition, we summarize the correlation among NLRP3 inflammasome activation in the liver-gut axis, liver injury, and intestinal barrier disruption in PBC and PSC. We summarize the differences in microbial and metabolic characteristics between PSC and IgG4-SC, and highlight the uniqueness of IgG4-SC. We explore the different roles of NLRP3 in acute and chronic cholestatic liver injury, as well as the complex and controversial crosstalk between various types of cell death in AILDs. We also discuss the most up-to-date developments in inflammasome- and pyroptosis-targeted medicines for autoimmune liver disorders.

## 1 Introduction

Autoimmune liver diseases (AILDs) are a group of chronic liver diseases caused by immune dysfunction (1), including autoimmune hepatitis (AIH), primary biliary cholangitis (PBC), primary sclerosing cholangitis (PSC), IgG4-related sclerosing cholangitis (IgG4-SC), and the so-called overlap syndrome (2). In AIH, hepatocytes undergo pathological alterations, with high serum levels of transaminase and IgG (or gamma globulin) and positive serum antinuclear and smooth muscle antibodies (3). Inflammation of interlobular bile ducts and tiny bile ducts is the primary hallmark of PBC (4). Liver function tests show intrahepatic cholestasis and a significant increase in serum IgM levels, positive serum antinuclear antibodies, and anti-smooth muscle antibodies (5). PSC lesions mainly occur in the intrahepatic bile duct, and in a few patients, the extrahepatic bile duct can be affected (6). Most patients also have ulcerative colitis (UC). IgG4-SC shares many primary clinical symptoms with PSC, such as multifocal biliary strictures, and is characterized by high serum IgG4 levels and lymphoplasmacytic infiltration of a significant number of IgG-positive cells (7). Symptoms from any two of the four disorders may be considered to reflect overlap syndrome. Immune-mediated liver disease is the result of innate and adaptive immune-mediated hepatocyte damage (8). The liver immune system is characterized by the predominance of innate components. AILDs is characterized by the loss of self-tolerance, which is caused by activation and proliferation of innate immune cells, especially macrophages, and autoreactive CD4 and CD8 T cells (9). Macrophages are thought to be a major source of inflammasomes and proinflammatory cytokines (10). The innate immune system supports the surveillance of pathogenic and aseptic damage caused by multicellular organisms. Pattern recognition receptors (PRRs) respond to pathogenic virulence factors, environmental toxins, and host-derived danger signals to initiate cytokine and chemokine production, thereby activating inflammasomes and subsequent pyrodeath that promote liver inflammation (11).

Pyroptosis, a type of programmed cell death, is crucial for defending the liver against infections (12). Inflammatory caspases and the gasdermin (GSDM) protein family are required for pyroptosis (13). GSDMs are a class of efficient factors that can create pores in the cell membrane during pyroptosis (14). Among the many molecules involved in pyroptosis, GSDMs play a crucial role. *GSDMA*, *GSDMB*, *GSDMC*, *GSDMD*, *GSDME*, and *PJVK* are six identified human paralogous genes. With the exception of PJVK, all GSDMs can create pores in the cell membrane, thus triggering pyroptosis (15). The inflammasome, a multiprotein signal transduction complex, assembles in the cytoplasm in response to danger signals produced by the host or a pathogen and activates caspase-1 (16). Activated caspase-1 promotes the transcription and expression of the inflammatory factors interleukin (IL)-1β and IL-18 and cleaves GSDMD, which results in the release of the active N-terminal domain. The N-terminal domain of GSDMD is a p30 fragment that is capable of forming pores in the cell membrane, thereby causing pyroptosis and inflammatory reactions (17). Inflammasome-mediated GSDMD-dependent pyroptosis is considered the canonical mode of pyroptosis, which is closely related to the inflammatory response and the severity of liver injury in AILDs and has been recognized as core components that drive immune-mediated pathology in hepatitis and liver injury (18).

This review addresses the links among inflammasomes, pyroptosis, and AILDs, as well as the similarities among these disease models, advancements, controversies, and the limitations of the current data. Furthermore, numerous studies have linked gut microbiota and the gut barrier to the etiology of liver disease (19). We conclude that there is a correlation among NLRP3 inflammasome activation in the liver-gut axis, liver injury, and intestinal barrier disruption in PBC and PSC, which may be closely related to the susceptibility of patients with PBC or PSC to inflammatory bowel disease. We also summarize the differences in microbial and metabolic characteristics between PSC and IgG4-SC, and highlight the uniqueness of IgG4-SC. We discuss the different roles of NLRP3 in acute and chronic cholestatic liver injury. We also explore the gaps in existing AILD-related research, such as the need to understand the pro-inflammatory and pro-pyroptotic mechanisms of NLRP6 inflammasomes associated with the gut-liver axis in AILDs, as well as the complex and controversial crosstalk between various types of cell death in AILDs.

## 2 Composition and classification of inflammasomes

**Figure 1:**
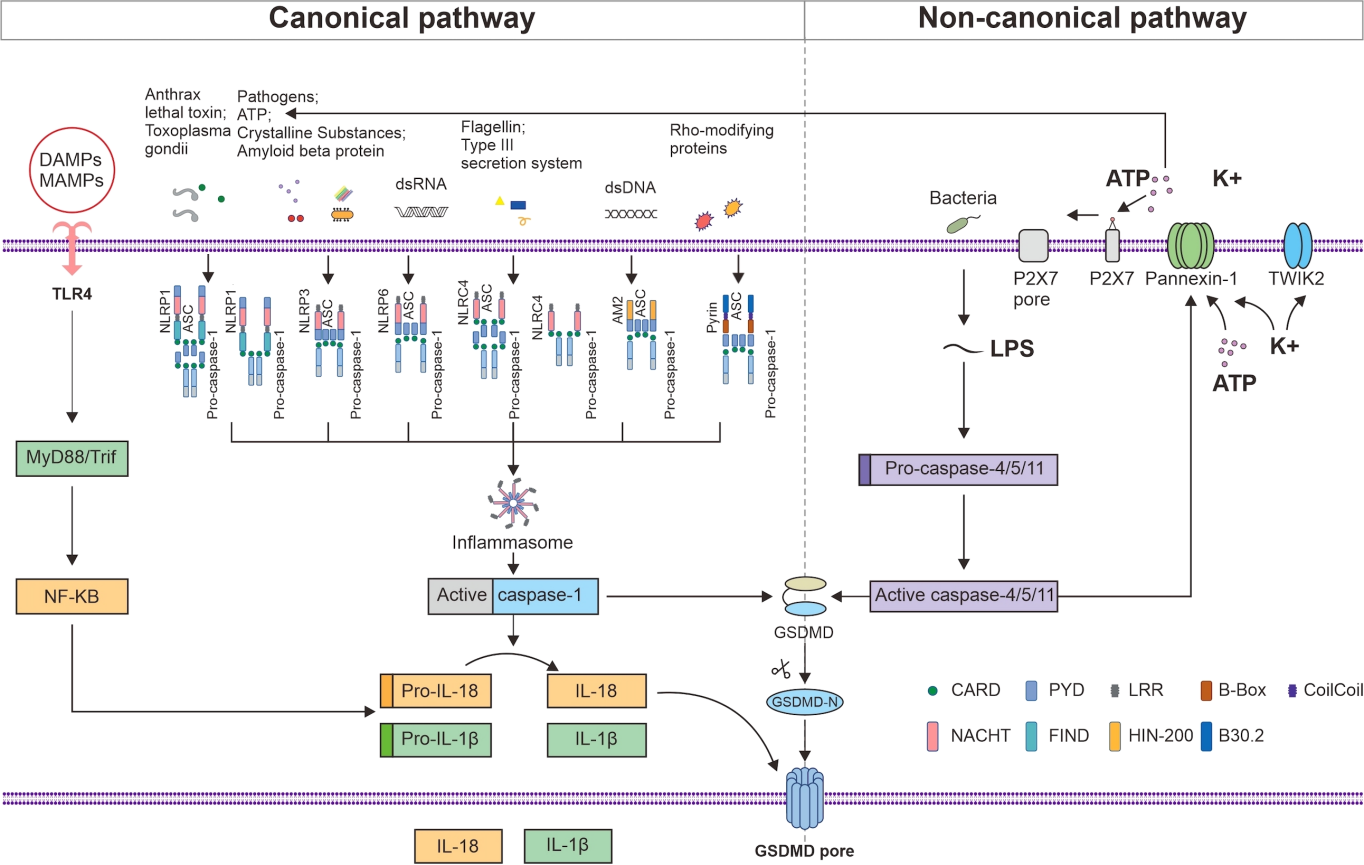
NLRP1, NLRP3, NLRP6, NLRC4, AIM2, and Pyrin can recruit procaspase-1 in an ASC-dependent manner, whereas NLRP1 and NLRC4 may also recruit procaspase-1 to the complex *via* CARD–CARD interactions. MAMPs and DAMPs are detected by TLR4, which induces the MyD88/NF-κB signaling pathway to produce pro-IL-1β and pro-IL-18. Activated caspase-1 promotes the maturation and secretion of IL-1β and IL-18 and cleaves GSDMD. The released active N-terminal domain of GSDMD causes membrane perforation and the release of the cell contents. LPS initiates pyroptosis *via* the noncanonical route by activating mouse caspase-4 and 5 or human caspase-11 to cleave GSDMD. The activated caspase-4, 5, and 11 cleave pannexin-1 and induce the release of ATP and K^+^, thereby activating the NLRP3 inflammasome. The efflux of K^+^ and ATP also activates P2X7, which forms a P2X7 pore in the cell membrane, promoting Ca^2+^ and Na^+^ influx. TWIK2 mediates K^+^ efflux in coordination with P2X7 and thus initiates P2x7-related pyroptotic cell death.

Two families of receptors make up the inflammasome sensor complex: the nucleotide-binding oligomerization domain-like receptors (NLRs) family, including NLRP1, 2, 3, 6, NLRC4 andNLRP12, and the HIN domain-containing (PYHIN) family, including absent in melanoma 2 (AIM2) and pyrin (20). Most inflammasomes consist of sensors (NLRs or PYHIN), caspase recruitment domains (CARD), and procaspase-1. NLRs consist of the following three components: ligand-binding N-terminal signaling domain, nucleotide binding oligomerization domain (NACHT), and nucleotide-binding C-terminal region containing a leucine-rich repeat (LRR) (21). Whether or not an NLR component requires the apoptosis-stimulating complex (ASC) adapter for assembly depends on the presence of a pyrin domain (PYD) or CARD in its N-terminus (22). AIM2 structurally contains an N-terminal PYD domain and a C-terminal hematopoietic interferon-inducible nuclear protein with a 200-amino acid repeat (HIN200) domain (23). Pyrin has two B-boxes, a PYD, a coil–coil domain, and a B30.2 domain (24). ASC structurally consist of PYD and CARD, whereas procaspase-1 has a CARD structure; therefore, it can promotes PYD and CARD homotypic interactions between sensors (NLRs or PYHIN) and procaspase-1 (25). Procaspase-1 can be recruited to complexes in two different ways: *via* the junction with ASC or *via* CARD–CARD interactions, which represents direct contact (26). All six inflammasome receptors can recruit procaspase-1 in an ASC-dependent manner, whereas NLRP1 and NLRC4 may also recruit procaspase-1 to the complex *via* CARD–CARD interactions (**Figure 1**). Inflammasomes are formed when PRRs, such as NLRs, on cells recognize microbe-associated molecular patterns (MAMPs) or endogenous damage-associated molecular patterns (DAMPs) and bind to caspase-1 and ASC, thus triggering the release of proinflammatory factors and GSDMD-mediated pyroptosis (27).

### 2.1 NLRP1 inflammasome

A C-terminal function-to-find domain (FIIND) is also present in NLRP1 but not in NLRP3 (**Figure 1**). Posttranslational proteolytic cleavage of the FIIND yields a heterodimer comprising the ZU5 and UPA subdomains, which are essential for NLRP1 activation *via* their noncovalent bonds and interaction with the remaining FIIND (28). According to a recent study, one kind of NLRP1 activation involves the modification and degradation of the N-terminal region of the *NLRP1* gene by anthrax lethal toxin and *Shigella flexneri* (29). The other kind of activation involves indirect activation by *Toxoplasma gondii* infection, inhibitors of dipeptidyl peptidases 8 and 9, and metabolic inhibitors (30). NLRP1 inflammasome activation is followed by caspase-1-dependent cytokine release, which promotes pyroptosis (31). In the livers of patients diagnosed with PBC or PSC cholestasis, the expression of NLRP1, caspase-1, and IL-1β was found to be increased (32). In addition to caspase-1, NLRP1 can also aggregate in a complex with caspase-5, but the role of caspase-5 is still controversial. Owing to the structural and functional differences between the human and mouse NLRP1 proteins, it is not always easy to investigate the function of the NLRP1 inflammasome in AILDs (33).

### 2.2 NLRP3 inflammasome

The NLRP3 inflammasome can be activated by both MAMPs and DAMPs, such as extracellular ATP, amyloid beta protein, and uric acid crystals (34). Lipopolysaccharide (LPS) and ectopic cytokines may also activate human caspase-4 and 5 or mouse caspase-11 to initiate pyroptosis, which in turn triggers ATP and K^+^ efflux and activates the NLRP3 inflammasome (35). NLRP3 oligomerizes through intercellular connections between NACHT domains and recruits ASC through homologous PYD–PYD interactions to initiate the formation of helical ASC filaments in response to stimulation. The assembled ASC recruits procaspase-1 through CARD–CARD interactions (**Figure 1**). The aggregated caspase-1 on ASC filaments splits at the p20/p10 juncture. The p20–p10 heterotetramer is released from ASC after further processing that involves CARD and p20. The activated caspase-1 cleaves GSDMD to trigger pyroptosis and amplify the inflammatory response by promoting the maturation and secretion of IL-1β and IL-18. NLRP3 complexes are critical in AILDs as well as in UC (36).

### 2.3 NLRP6 inflammasome

NLRP6 has the ability to directly bind double-stranded (ds) RNA, which can recognize microbial metabolites (37). Liquid–liquid phase separation occurs when NLRP6 interacts with dsRNA (38). Moreover, lipoteihoic acid, a known NLRP6 ligand, can promote NLRP6 liquid–liquid phase separation, and the RNA helicase DHX15 can form a complex with NLRP6 and dsRNA (39). NLRP6 protein is mostly expressed in the intestine, followed by the liver (40). In intestinal goblet cells, Toll-like receptor (TLR) ligands activate the myeloid Differentiation Factor 88 (MyD88)–reactive oxygen species (ROS) pathway and NLRP6 inflammasome. By forming a helical structure, NLRP6 attracts ASC and then recruits caspase-1 to cleave GSDMD, which promotes IL-18 production and leads to pyroptosis (41). NLRP6 inflammasome secretes IL-18, which leads to production of antimicrobial peptides to maintain intestinal homeostasis. However, when intestinal homeostasis is disrupted, NLRP6 expression is increased in macrophages and IL-18 secretion promotes intestinal inflammation (42). The gut–liver axis links disturbances in the intestinal microbial equilibrium to autoimmune liver disorders (43). NLRP6 is a protective factor against the development of nonalcoholic fatty liver disease (NAFLD) and has been found to play a crucial role in inflammatory and immunological responses (44). Reports have suggested that the *NLPR6* gene is a candidate tumor suppressor gene (45). This gene has been shown to be capable of inducing pyroptosis in hepatocellular carcinoma and is linked to higher levels of immune cell infiltration, both of which are associated with a more favorable prognosis. A negative association has been revealed between NLRP6 expression and alpha-fetoprotein (AFP) >400 ng/mL and mortality in Asian patients (46). Compared with liver biopsy samples from healthy controls, fibrotic and cirrhotic livers have lower levels of Nlrp6 expression (44). Therefore, the NLRP6 inflammasome is an essential component in both the upkeep of the gut microbiota and the protection of the liver against inflammatory damage.

### 2.4 NLRC4 inflammasome

NLRC4 consists of the CARD, NACHT, and LRR domains and either dependently or independently activates caspase-1 (**Figure 1**). The human neuronal apoptosis inhibitory protein (NAIP) senses bacterial flagellin and type III secretion system components to form NAIP/NLRC4 inflammasomes (47), which are formed by oligomerization with NLRC4 adapters to recruit and activate caspase-1 (48). Patients with NAFLD have increased levels of tumor necrosis factor (TNF) expression and NLRC4 inflammasome activation, which results in a rise in the synthesis of IL-18 and IL-1β and causes pyroptosis (49). The NLRC4 inflammasome has been shown to be crucial in bacterial infections in the liver, and NLRC4-mediated IL-1β release has been linked to liver inflammation.

### 2.5 AIM2 inflammasome

AIM2 is able to detect dsDNA derived from the host or a pathogen, which allows it to recruit ASC and procaspase-1 for inflammasome formation (**Figure 1**). Kupffer cell inflammation is exacerbated when AIM2 is absent in hepatocellular cancer (50). Reports have suggested that AIM2 may be related to hypoxia/reoxygenation-mediated hepatocyte pyroptosis because interference with *AIM2* gene expression could reduce L02 cell pyroptosis caused by hypoxia/reoxygenation by suppressing caspase-1, IL-1β, and IL-18 levels (51). Additionally, AIM2 has been shown to have its own unique expression pattern in chronic viral hepatitis (52).

### 2.6 Pyrin inflammasome

The *MEFV* gene encodes a protein called pyrin, which has two B-boxes, a PYD, a coil–coil domain, and a B30.2 domain (24). Pyrin is stimulated by Rho-modifying proteins and recruits caspase-1 in an ASC-dependent manner (**Figure 1**) (53). Mouse pyrin lacks the B30.2 domain. Moreover, the bile acid analog BAA485 was shown to activate the pyrin inflammasome pathway in immune and intestinal epithelial cells (54). However, whether endogenous bile acids from the gut microbiota, which is intimately associated with the inflammatory response in AILDs, can trigger pyrin inflammasome formation has not been determined.

## 3 Inflammasome-mediated pyroptosis

Recently, pyroptosis has been reclassified as gasdermin-dependent cell death after the pore-forming protein GSDMD was discovered to be a caspase-1, 4, 5, and 11 substrate necessary to mediate pyroptosis (55). Inflammasome-mediated GSDMD-dependent pyroptosis is considered to involve a canonical signaling pathway. As pyroptosis effector molecules, members of the GSDM protein family have a crucial function. In humans, six GSDM genes have been identified, namely, *GSDMA*, *GSDMB*, *GSDMC*, *GSDMD*, *GSDME* (also known as *DFNA5*), and *PJVK* (also known as *DFNB59*). With the exception of PJVK, all GSDMs consist of a C-terminal inhibitory domain and an N-terminal effector domain. Hydroxylation of the C-terminal domain frees the N-terminal domain to generate pores in the cell membrane by binding to lipid components (56).

### 3.1 GSDM protein family

In mice, three isoforms of Gsdma (Gsdma1–3) and four isoforms of Gsdmc (Gsdmc1–4) are present (57), while Gsdmb is not observed. Streptococcus A secretes the protease streptococcal exotoxin B (SpeB), which can directly cleave GSDMA to activate pyroptosis (58). Recent studies have found that GSDMA-deficient mice are susceptible to streptococcal infection in a SpeB-dependent manner (59, 60). Caspase-3, 6, and 7 have the ability to break down GSDMB. GSDMB promotes the activation of caspase-4 by binding to the CRAD domain of caspase-4, which may be another pathway of pyroptosis (61). Granzyme A (GzmA) can cleave GSDMB expressed in gastrointestinal epithelial tumors and lead to the pyroptosis of target tumor cells (62). Under hypoxia, programmed cell death 1 ligand 1 (PD-L1) and phosphorylation signal transducer and activator of transcription 3 (p-STAT3) work together to control the transcription and cleavage of GSDMC, which ultimately results in a switch from TNF-activated caspase-8-mediated apoptosis to pyroptosis (63). GSDMC can be used as an additional predictive factor for hepatocellular carcinoma (64). Furthermore, the expression levels of mouse GSDMC are positively correlated with the metastatic ability of B16 melanoma cell lines (65). However, knockdown of GSMDC attenuates the proliferation of colorectal cancer cell lines (66). Thus, whether GSMDC acts as a pro-tumor regulator or an anti-tumor regulator in tumor development remains to be verified by more experiments and trials. The mechanism of pyroptosis, which is mediated by GSDMD as a star molecule, is evident. GSDMD can mediate pyroptosis by two primary methods. Canonical inflammasome-triggered pyroptosis is caused by caspase-1 cleavage of GSDMD, while noncanonical inflammasome-triggered pyroptosis is caused by caspase-11 (or caspase-4 or 5) cleavage of GSDMD (67). In addition, transforming growth factor (TGF)-β-activated kinase 1 inhibition has been reported to activate caspase-8 by ligating and oligomerizing cell surface death receptors, which in turn causes GSDMD-dependent pyroptosis (68). Macrophages, dendritic cells, and neutrophils, which are also known as hyperactivated cells, are able to endure inflammasome-activated cleavage of GSDMD without membrane rupture and pyroptosis in some circumstances (69). In a granzyme-mediated pathway, chimeric antigen receptor (CAR) T cells release GzmB, which can cleave GSDME directly or indirectly by activating caspase-3, which results in pyroptosis (70), thereby enhancing the capacity of CAR T cells to kill target cells (71). GSDME-induced pyroptosis of cochlear hair cells can lead to hearing impairment. Furthermore, PJVK is highly similar to GSDME, and the gene is detected in neurons of the inner ear and auditory system, which has been reported to be associated with deafness (72).

### 3.2 Canonical and noncanonical signaling pathways of inflammasome activation to drive pyroptosis

In the canonical pathway, TLR4 is responsible for recognizing MAMPs and DAMPs, activating nuclear factor kappa-B (NF-κB), inducing the downstream MyD88/NF-κB signaling pathway, and causing macrophages to produce pro-IL-1β and pro-IL-18 (**Figure 1**) (73). In response to the activation of intracellular threat signals, NLRs or PYHIN bind to procaspase-1 and ASC, causing inflammasome formation and activating caspase-1. Activated caspase-1 promotes the transcription and expression of the inflammatory factors IL-1β and IL-18, which are then released into the extracellular space to recruit inflammatory cells and expand the inflammatory response (74). Furthermore, GSDMD is cleaved by activated caspase-1, which results in the removal of the inhibitory C-terminal domain and release of the active N-terminal domain. The N-terminal domain of GSDMD is a p30 fragment that is capable of forming pores in the cell membrane (75), thereby causing membrane perforation, cell rupture, and inflammatory reactions (**Figure 1**).

In the noncanonical pathway, intracellular LPS activates human caspase-4 or 5 or mouse caspase-11. The activated caspases subsequently catalyze the cleavage of GSDMD and initiate pyroptosis to enhance intracellular K^+^ exocytosis (76). Additionally, activated caspases 4, 5, and 11 cleave pannexin-1, induce ATP release, activate P2X purine receptor 7 (P2X7) to form P2X7 pores in the cell membrane, promote Ca^2+^ and Na^+^ influx, and then coordinate with the two-pore domain K channel (TWIK2), which mediates K^+^ efflux (77), thus triggering P2x7-related pyroptotic cell death (78). The efflux of K^+^ and ATP activates the assembly of the NLRP3 inflammasome and activates caspase-1, which leads to the secretion of IL-1β and IL-18 (**Figure 1**).

## 4 Inflammasomes and pyroptosis in AILDs

### 4.1 Inflammasomes and pyroptosis in AIH

AIH is a group of conditions in which the body’s immune system attacks its own liver cells, leading to chronic liver damage (79). Previous studies have suggested that AIH involves T cells (in a T cell receptor independent manner) and activation of natural killer T (NKT) cells mediated by cytokines (80). NKT cells in AIH mouse models express costimulant OX40 and high levels of caspase-1 (81). OX40 can reduce the immunosuppressive activity of T cells (Tregs), thereby further amplifying the T cell activation effect (82). Activation of caspase-1 leads to maturation and secretion of IL-1β and GSDMD-mediated pyroptosis (83). A recent study found that inflammasome-mediated pyroptosis and massive cytokine production affect the inflammatory response in AIH and the degree of inflammation in liver injury, which is thought to be one of the key events in AIH progression (84).

#### 4.1.1 NLRP3 inflammasome-mediated pyroptosis in AIH can be counteracted by IL-1 receptor antagonists

Concanavalin A (ConA)-induced hepatitis, as a mature experimental model for immune-mediated liver injury, can mimic human AIH to a certain extent (85). In ConA-induced hepatitis, the expression of the NLRP3 inflammasome and the levels of activated caspase-1, IL-1β, and lactate dehydrogenase are elevated in the blood. Staining for dead cells and western blot analysis revealed that pyroptosis was the primary mode of death for ConA-induced mouse hepatocytes (86). Furthermore, the NLRP3 inflammasome and its downstream products were shown to be highly expressed in hepatocytes and, to a lesser extent, in immature hepatocytes (19).

**Figure 2:**
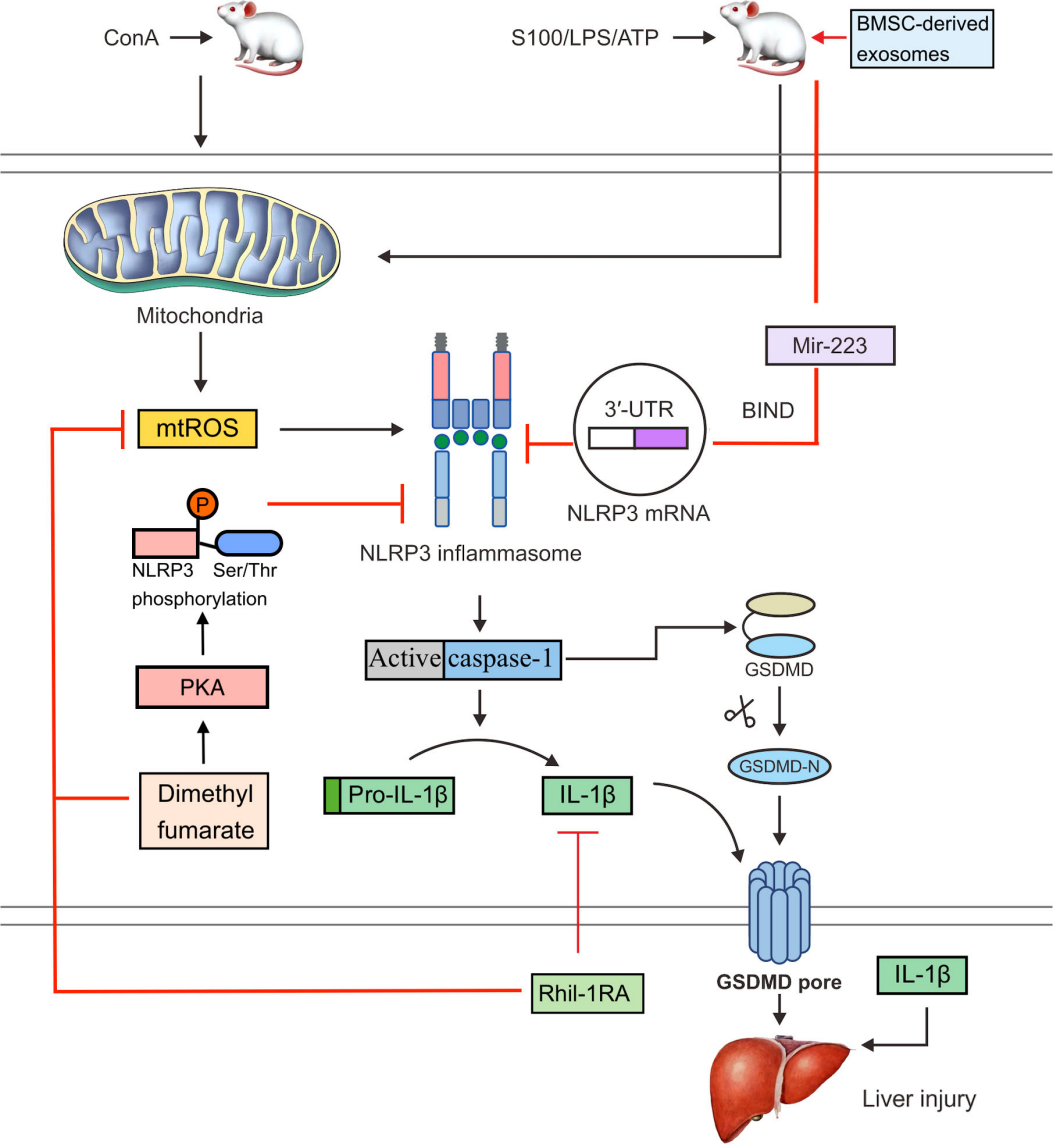
In ConA-induced mice, mtROS can activate the NLRP3 inflammasome. Activated caspase-1 cleaves GSDMD and releases an active GSDMD N-terminal domain, which forms pores in the cell membrane and triggers pyroptosis. Activated caspase-1 also cleaves pro-IL-1β to IL-1β. An rhil-1ra can eliminate mtROS, reduce NLRP3 inflammasome activation and pyroptosis, suppress the expression of IL-1β, and alleviate ConA-induced hepatitis. Dimethyl fumarate can reduce mitochondrial damage and the production of mtROS. It can also enhance the PKA signaling pathway and increase the phosphorylation of NLRP3 on Ser/Thr at PKA-specific sites, thereby inhibiting the activation of NLRP3 inflammasome. Bmsc-derived exosomes bind to the 3′UTR of the *Nlrp3* mrna through exosomal miR-223, which interferes with protein translation and inhibits NLRP3 inflammasome activation, thereby reversing hepatocyte injury in the S100- or LPS/ATP-induced mouse AIH model.

However, research has shown that recombinant human IL-1 receptor antagonists (rhil-1RAs) can suppress NLRP3 inflammasome activation and IL-1β production (87). The NLRP3 inflammasome can be activated by MAMPs and DAMPs, and mitochondria-derived ROS (mtROS) are thought to be critical factors that promote this activation (88, 89). An rhIl-1RA was demonstrated to significantly reduce pyroptosis by lowering ROS levels in ConA-induced mice and the production of NLRP3, active caspase-1, and IL-1β in hepatocytes (**Figure 2**) (90). These results suggest that rhil-1RA has the ability to eliminate ROS, reduce NLRP3 inflammasome generation, prevent pyroptosis, and compete with IL-1β to lower the severity of ConA-induced hepatitis.

#### 4.1.2 Inhibitory effect of BMSC-derived exosomes on NLRP3

Endocytic miRNAs are single-stranded non-coding RNAs that range in length from 19 to 24 nucleotides and regulate NLRP3 inflammasome formation (91). Various miRNAs, including miR-223, miR-22, and miR-7, can control *NLRP3* mRNA expression (92). In particular, miR-223 binds to the 3′-untranslated region (3′UTR) of the *NLRP3* mRNA and blocks protein translation at this point (93). Both miR-22 and miR-7 have been shown to modulate NLRP3 activation during inflammation, which may have beneficial effects (94). miR-223 is highly expressed in bone marrow mesenchymal stem cells (BMSCs). Bmsc-derived exosomes effectively reversed S100- or LPS/ATP-induced AIH and hepatocyte damage in a mouse model and also downregulated the expression of NLRP3 and reduce caspase-1 levels (**Figure 2**) (95). A possible explanation is that exosomal miR-223 from BMSCs inhibits NLRP3 inflammasome activation.

#### 4.1.3 Protective effects of GSDMD in AIH

GSDMD plays the role of the executioner in pyroptosis as a substrate for caspase-1 and caspase-11, both of which are necessary to mediate pyroptotic cell death. The role of GSDMD in the many pathological forms of liver disease has been the subject of debate. It has been revealed that the N-terminal domain of GSDMD is responsible for some of the negative consequences observed in metabolic liver illnesses, such as NAFLD. Some studies have found that inhibition of GSDMD protects mice from this type of liver disease (96). In contrast, GSDMD deficiency was found to cause more portal vein and lobular inflammation, a wider hepatic conjunctival necrotic area, higher serum transaminase levels, and more extensive hepatocyte apoptosis after ConA induction (86). In addition, liver injury was aggravated in GSDMD-deficient mice and was accompanied by intestinal barrier destruction. GSDMD-deficient mice showed significantly increased expression of Lps-binding protein, which is known to be an indicator of LPS exposure, and upregulated expression of TLR4 and CD14 (97). Owing to increased intestinal permeability, bacterial LPS can be carried to the liver *via* the portal vein, wherein it can bind to TLR4 on the surface of hepatic Kupffer cells and trigger an immunological response in the liver (98). The mechanism may be related to the fact that GSDMD knockdown inhibits pyroptosis but promotes apoptosis, indicating the tampering effect of GSDMD between different types of cell death (99). The role of GSDMD in AIH is significant and complex and worthy of further study.

### 4.2 Inflammasomes and pyroptosis in PBC

The pathophysiology of PBC is multifactorial and involves the immune system, aberrant bile salt production, impaired biliary transport, and programmed cell death of cholangiocytes (100). Innate immune cells and adaptive immune cells together causes damage to the PBC small bile duct (101). Innate immunity focuses on damage to self-reactive CD4 T cells, CD8 T cells, and Tregs. CD8 T cells mainly infiltrate the portal vein and damage small bile duct of the recipient mice, which present typical PBC symptoms. Tregs are functionally deficient, which impairs their immunosuppressive function (102). In PBC, the B-cell activator BAFF is significantly correlated with the cholestatic enzyme level, bilirubin content, AMA titer, and disease stage (103). Recently, the activation of inflammatory bodies in innate immunity has been identified as an important factor in the progression of liver inflammation (104). In peripheral blood of patients with PBC, monocytes are more sensitive to external stimuli. Under the stimulation of MAMPs, TLRs mediate the maturation and secretion of pro-inflammatory cytokines, including IL-1 and IL-18, and activate inflammasome-mediated pyroptosis, which leads to the release of inflammatory factors outside the cell and expands the inflammatory response (105).

#### 4.2.1 Activation of the NLRP3 inflammasome by galectin-3 (Gal3) drives autoimmune cholestatic liver injury

The pleiotropic lectin Gal3 is an important regulator of inflammatory signaling in hepatic macrophages and contains C- and N-terminals (106). Gal3 expression is upregulated during differentiation of human monocytes to macrophages under physiological conditions and in macrophages and stellate cells involved in liver disease under pathological conditions (107). It is well established that DAMPs are capable of activating the NLRP3 inflammasome, and Gal3 is considered a DAMP molecule (108). The activation of the inflammasome is induced by the interaction of the N-terminal domain of Gal3 with NLRP3. Gal3 plays a significant role in the activation of inflammasomes, thereby leading to the development of PBC (109).

**Figure 3:**
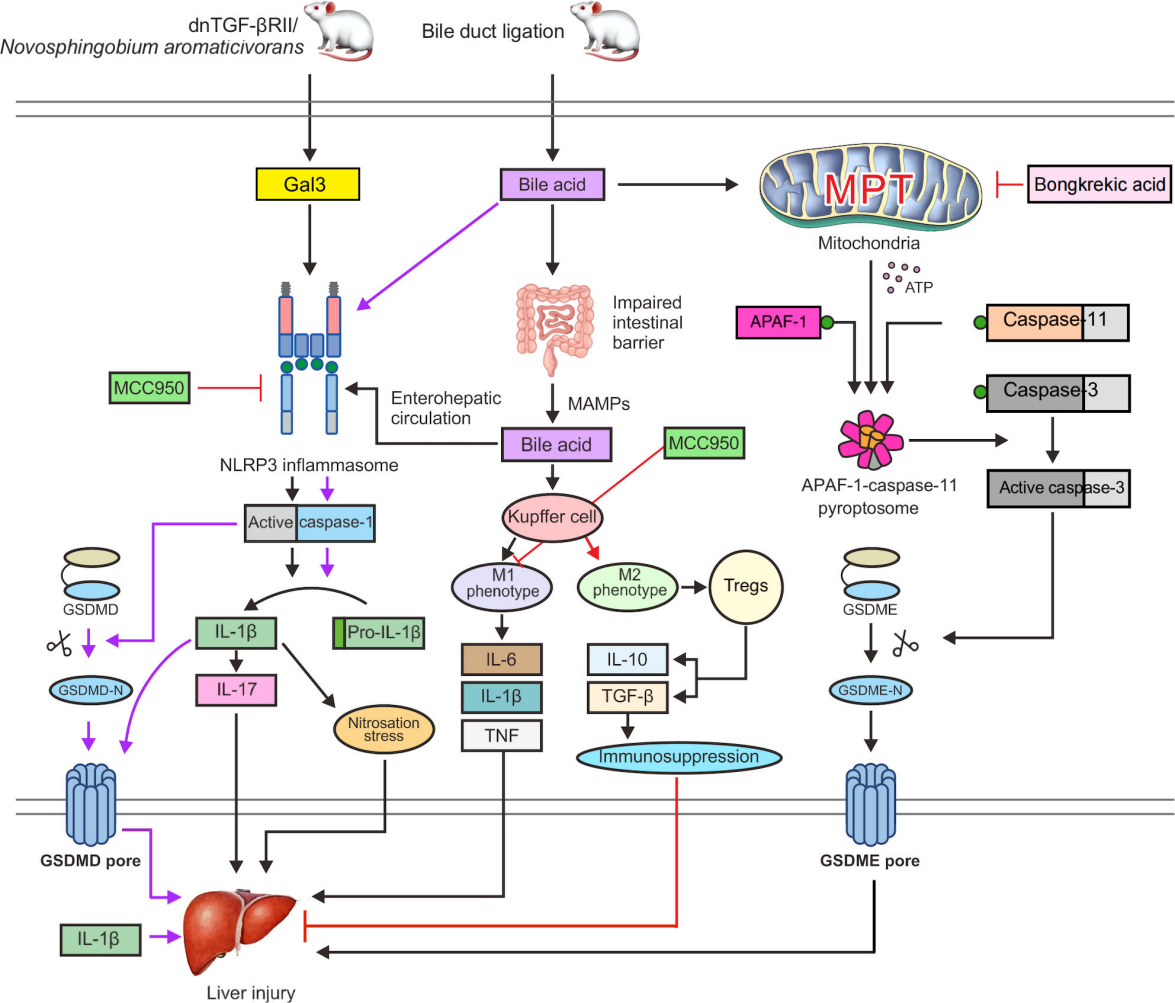
Gal3 activates the NLRP3 inflammasome and caspase-1 in dnTGF-βRII mice or PBC mice infected with *Novosphingobium aromaticivorans*, which can stimulate the release of IL-1β and GSDMD cleavage to induce pyroptosis. Nitrosation stress caused by IL-1β can exacerbate liver damage. Additionally, IL-1β can cause macrophages to generate IL-17 and promote liver fibrosis. Bile acids can cause NLRP3 inflammasome activation and caspase-1 cleavage of GSDMD in BDL mice, which results in pyroptosis. The intestinal microecology and permeability are also impacted by bile acids, which can cause MAMPs to enter the interhepatic circulation through the enterohepatic axis, increasing the exposure of the liver to endotoxins. Toxic bile acids further activate NLRP3 inflammasome assembly and cause Kupffer cells to develop the M1 phenotype, leading to the production of proinflammatory cytokines, including IL-6, TNF, and IL-1β, which support liver fibrosis and damage. MCC950 can directly prevent the activation of the NLRP3 inflammasome. Furthermore, MCC950 can cause macrophage polarization to the M2 phenotype, which activates Tregs to release IL-10 and TGF-β. These factors can boost immune suppression and minimize liver damage. In addition, bile acids can trigger the MPT state and the release of ATP into the cytoplasm, which promotes the fusion of APAF-1 and caspase-11 to form an APAF-1/caspase-11 pyroptosome. Caspase-3 is then triggered to cleave GSDME, which leads to the formation of GSDME pores, thereby exacerbating liver damage and leading to pyroptosis. Bongkrekic acid can prevent the ANT-mediated MPT, thus preventing GSDME-dependent pyroptosis.

In a dominant-negative transforming growth factor-β receptor type II (dnTGF-βRII) mouse model of spontaneous PBC formation (107), recombinant Gal3 boosted NLRP3 inflammasome activation and was significantly expressed in dendritic cells and macrophages. Nitrosation stress is another potential mechanism of chronic liver damage, and the build-up of nitrosated tyrosine has been linked to bile duct injury in PBC (110). IL-1β can induce nitrosation stress; therefore, the Gal3/NLRP3 inflammasome/IL-1β signal transduction pathway may be an important pathway involved in the pathogenesis of the disease (**Figure 3**). In addition, IL-1β can activate macrophages to produce IL-17 and promote liver fibrosis (111). IL-17 is widely expressed in portal regions, with evident inflammatory cell infiltration in liver tissues of patients with AIH (112), PBC (113), and overlap syndrome (114), and it is considered a significant proinflammatory and profibrotic agent in the liver (115, 116). Accordingly, hepatic macrophage activation of Gal3/NLRP3 inflammasome/IL-17 signaling may be an additional significant pathogenic mechanism of PBC fibrosis (117). A previous study showed that Gal3 depletion in dnTGF-βRII mice decreases IL-1β and IL-17 generation and alleviates bile duct inflammation (118). In a mouse model of PBC caused by infection with *Novosphingobium aromaticivorans*, Gal3-deficient animals did not develop PBC following surgery (119). Weak activation of the NLRP3 inflammasome may be the cause, thus leading to underdeveloped dendritic and other immune cells. In conclusion, early proinflammatory damage in PBC is dependent on Gal3-mediated NLRP3 inflammasome activation. To treat PBC and other chronic cholestatic liver illnesses, blocking signal transduction pathways with Gal3 or NLRP3 inhibitors might be an option.

Studies have shown that Gal3 mediates noncanonical pyroptosis by facilitating LPS-induced oligomerization and activation of caspases-4 and 11 as well as the cleavage of GSDMD in cells (120). Cholestasis in PBC is known to result in an impaired intestinal barrier, increased intestinal permeability, and transfer of MAMPs, such as LPS and microbial RNA, to the liver *via* the interhepatic circulation (121). This key mechanism for fighting bacterial infections may provide opportunities for new therapeutic interventions. However, it is still unknown whether gut microorganisms can cause pyroptosis through the hepatointestinal circulation in PBC.

#### 4.2.2 Bile acids act on the hepatic immune system, biliary tract, and gut microbiota through the interhepatic circulation

Free fatty acids and endotoxins may flow into the portal circulation and hepatic sinusoids as a result of dysfunction of the intestinal epithelial barrier, which may then result in liver damage and inflammation (122, 123). Endotoxins have been shown to enhance the sensitivity of hepatocytes to cell death in response to bile acid challenge (124). Increased exposure to hepatic endotoxins is caused by the promotion of intestinal leakage by macrophages and changes in the gut microbiota after activation of inflammasomes during cholestasis (125). In bile duct ligation (BDL) mice, toxic bile acids further activate NLRP3 inflammasome assembly and cause Kupffer cells to develop the M1 phenotype, leading to the production of proinflammatory cytokines, including IL-6, TNF, and IL-1β, which aggravate liver fibrosis and damage (126) (**Figure 3**).

Changes in circulating bile acids have been found to be closely related to disease progression, and patients with PBC show bile acid-related intestinal dysregulation (127). Through the interhepatic circulation of bile acids, the liver can impact intestinal homeostasis and absorption by changing the quantity of the gut microbiota and the permeability of the intestinal mucosa (128). Some potentially helpful bacteria, such as *Ruminococcus bromii*, were found to be lacking in the gut microbiota of patients with PBC, whereas pathogens such as *Proteobacteria*, *Enterobacteriaceae*, *Streptococcus*, and *Klebsiella* were found to be abundant (129). Among ursodeoxycholic acid nonresponders, fecal bacteria show a lower relative abundance (130). Patients with advanced fibrotic PBC have higher levels of acetate as well as short-chain fatty acids in their feces. Therefore, decreased bacterial abundance in the feces may be used to predict the prognosis of patients with PBC.

#### 4.2.3 Induction of pyroptosis by apoptosis protease activating factor 1 (APAF-1)/Caspase-11/GSDME

Pyroptosis induction by GSDMD and GSDME was detected in liver tissues of BDL mice (131). In BDL mice, caspase-1 is activated by bile acids and cleaves GSDMD to generate the N-terminal p30 fragment required for pore formation during cell lysis. Surprisingly, bile acids activated caspase-11, although not in the same way as LPS did. Bile acids cannot break GSDMD to produce the p30 fragment; instead, the caspase-3 moiety cleaves GSDMD to an inactive p43 fragment. In human hepatocellular carcinoma (HepG2) cells and BDL mice, bile acids induce persistent mitochondrial permeability transition (MPT), a state of loss of the mitochondrial inner membrane integrity, allowing free permeation of small solutes (132). Upon MPT stimulation, ATP is released into the cytoplasm to combine APAF-1 and caspase-11 into an APAF-1/caspase-11 pyroptosome at a ratio of 7:2, and then caspase-3 is activated to cleave GSDME and trigger GSDME-dependent pyroptosis. Under MPT conditions following bile acid stimulation, APAF-1 was found to selectively recruit and activate caspase-4 (**Figure 3**) (133). However, in Apaf-1 knockdown human HepG2 cells, bile acids were unable to activate GSDME-dependent pyroptosis. Thus, inhibiting APAF-1 inflammasome might be effective therapeutic approaches for preventing inflammation-induced liver damage.

Recent studies have reported that doxorubicin and a moderate concentration of choleric acid (50 mM) facilitate mitochondrial outer membrane permeabilization and initiate apoptosis by accelerating the assembly of an Apaf-1/caspase-9 apoptosome (134). In contrast, a high concentration of bile acid (200 mM) promotes Apaf-1/caspase-11 pyroptosome production and MPT-triggered pyroptosis. Therefore, the crosstalk between various types of cell death remains very complex and controversial.

### 4.3 Inflammasomes and Pyroptosis in PSC

PSC represents a robust cellular response in the epithelium of the bile duct, and it results in persistent inflammation and fibrosis that leads to intrahepatic or extrahepatic bile duct stenosis and cholestasis. PSC is intimately linked to UC. Approximately 70% of patients with PSC have inflammatory bowel disease (IBD), and 87% of these individuals also have UC (135). Recruitment of gut-derived memory T cells to the liver in regulatory immunity is thought to be a driver of PSC liver inflammation (136). CD8 T cells in enteric-associated lymphoid tissue induce immune-mediated cholangitis in mice (137). Liver CD8 T lymphocytes promote PSC biliary tract injury and fibrosis in mice, and their proliferation is controlled by liver Tregs, which are regulated by IL-2 signaling pathways (138). An increased frequency of T cells secreting interferon (IFN)-γ was found in the colon of PSC-UC patients compared with UC (139). IFN-γ in PSC mouse models altered the phenotype of CD8 T lymphocytes and NK cells in the liver, resulting in increased cytotoxicity (140). Mounting evidence from epidemiological and clinical research has shown that infection influences the development of PSC (141). Innate immunity after infection can be caused by a variety of mechanisms, including DAMPs and MAMPs, which are bound by PRRs, and these mechanisms induce the secretion of inflammatory cytokines, activate inflammasome and pyroptosis, and induce a range of immuno-inflammatory responses and programmed cell death (142).

#### 4.3.1 Gut–Liver Axis and Microbiota in PSC

IBD and PSC are closely connected, particularly in the populations of northern Europe, where up to 80% of patients with PSC also have IBD (143). The gastrointestinal tract communicates with the liver *via* portal circulation. Nutrients, antigens, hormones, and other substances all serve as signals. AILDs are associated with the microbiota composition through aberrant immune system activation, primarily *via* the gut–liver axis (144). Innate immune activation of bile duct cells is strongly associated with IBD, and abnormal amounts of LPS accumulate in bile duct cells in patients with PSC compared to other cholestatic diseases (145). Increased intestinal permeability leads to the flow of MAMPs into the systemic circulation and stimulates inflammation. MAMPs originating in the gastrointestinal tract, such as LPSs or microbial RNA, can enter the portal circulation and travel to the liver, causing hepatic inflammation and fibrosis in the liver (121).

Multiple studies have found that persons with PSC have an altered gut microbiota, which is marked by a decline in microbial diversity and a rise in the quantity of potential pathogens (146). Stool samples from patients with PSC are rich in the genus *Veillonella* and show increased relative abundances of *Enterococcus*, *Streptococcus*, and *Lactobacillus (* 147). Another unique finding is a marked reduction in bifidobacteria, which is associated with a failure to alleviate liver inflammation. Previous studies have identified *Enterococcus faecalis* as a potentially pathogenic bacterium that grows significantly in the bile duct of patients with PSC (148). According to research on patients with PSC, a higher level of circulating vascular adhesion protein 1 enhances the adherence of gut-derived lymphocytes to hepatic endothelial cells, which is related to poor outcomes in PSC patients (147). These findings shed light on the significance of the microbiota in the gut in relation to PSC.

#### 4.3.2 Dysregulation of intestinal microbiota promotes liver disease progression through nlrp3 in multi-drug resistant gene 2 (*Mdr2* ^−/−^) mice

**Figure 4:**
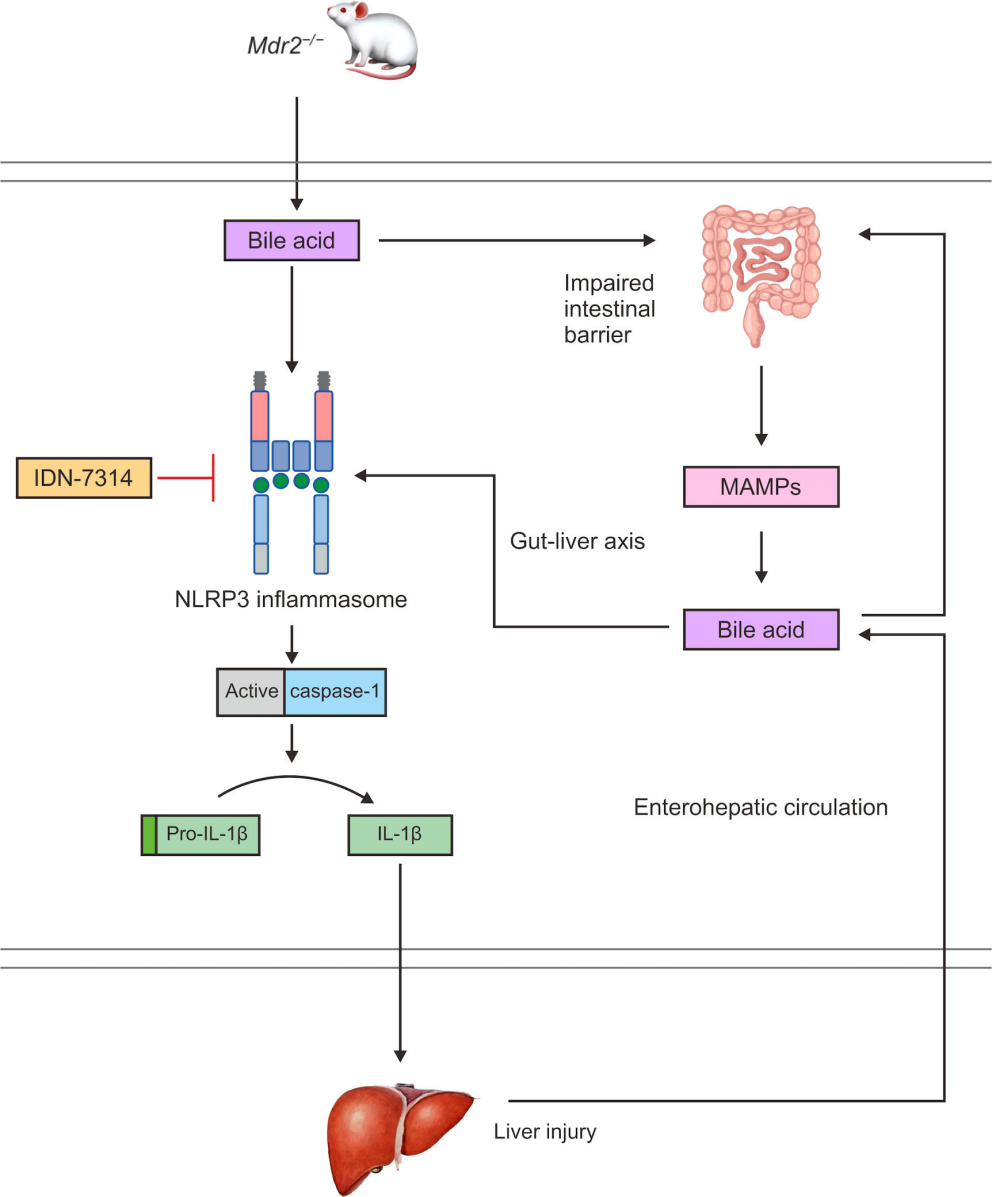
In *Mdr2* ^−/−^ mice, bile acids can activate the NLRP3 inflammasome and caspase-1 to cleave pro-IL-1β to IL-1β, promoting an inflammatory response and liver injury. Bile acids also damage the intestinal mucosal barrier and increase intestinal permeability, which can cause MAMPs to enter the interhepatic circulation through the enterohepatic axis, increasing the exposure of the liver to endotoxins. Toxic bile acids further activate NLRP3 inflammasome assembly and promote the maturation and secretion of IL-1β, thereby expanding the inflammatory response and aggravating liver injury. In turn, the liver can affect intestinal homeostasis through the interhepatic circulation of bile acids.

A frequently employed model for human PSC is the *Mdr2* ^−/−^ mouse. This model features that mimic human PSC pathology, including biliary damage, inflammation, and hepatic fibrosis (149). Immunohistochemistry analyses of the liver of *Mdr2* ^−/−^ mice indicated a large increase in NLRP3-positive cells and showed that cholestatic liver damage in *Mdr2* ^−/−^ mice was caused by considerable inflammatory activation (150). Mdr2-related cholestasis could induce dysregulation of the intestinal microbiota, and toxic bile acids enter the portal vein to activate the NLRP3 inflammasome and aggravate liver injury (**Figure 4**) (151). Changes in the plasma bile acid profiles in patients with PSC have been documented and clinically associated with hepatic decompensation. In addition, *Mdr2* ^−/−^ animals exhibit intestinal barrier failure as well as enhanced bacterial translocation, which boosts the activation of the NLRP3 inflammasome within the gut–liver axis (143). According to these findings, intestinal dysregulation caused by cholestasis is believed to have a direct influence on the course of liver disease. *Mdr2* ^−/−^ mice have higher levels of the caspase-11 mRNA than normal mice (152). Caspase-11 can cleave GSDMD, and it represents a key molecule involved in pyroptosis and may provide opportunities for new therapeutic interventions. However, experimental studies have not verified typical and atypical pyroptosis in PSC.

#### 4.3.3 Bidirectional role of NLRP3 in PSC

Bile acids and other regulatory molecules have been shown to induce the activation of the NLRP3 inflammasome (153). This causes the secretion of IL-1β, thus leading to transinflammatory liver injury and playing a role in reactive cholangiocyte inflammation in patients with PSC (154). In PSC mouse models fed with 3, 5-dioxy-carboxyl, 1, 4-dihydrogen collidine (DDC) and in human PSC, NLRP3 inflammasomes are increased in reactive bile duct cells, leading to IL-18 secretion and epithelial functional barriers of bile duct cells, thereby affecting the development of bile duct disease (155). However, experimental studies have reported contradictory results, indicating that bile acids induce NLRP3 expression and NLRP3-deficient mice show cholestatic liver injury and aggravation of fibrosis (156). Similarly, it has been suggested that NLRP3 inflammasome activation has a certain protective effect in a PSC mouse model. Moreover, cholangitis caused by NLRP3 deficiency had a more severe clinical nature, as shown by the bile duct damage, larger inflammatory lesions, and elevated levels of IL-6 and CXCL10 (157). Furthermore, NLRP3 depletion resulted in a hyper-IL-17 response, which was manifested as IL-17-independent exacerbation of the disease (84). Interestingly, NLRP3 plays different roles in the course of illness between acute and chronic cholestatic liver damage. A reduction in IL-18 expression was observed in the acute stage of injury in mice lacking NLRP3, although an increase in apoptosis was noted. However, wild-type mice showed increased IL-18 levels in the acute phase and decreased IL-18 levels in the chronic phase (158).

These results indicated a dual function for the NLRP3 inflammasome in cholestatic liver damage. During acute cholestatic liver damage, the NLRP3 inflammasome may have a protective function by blocking some of the pathways that lead to cell death. On the other hand, during persistent cholestatic liver damage, the NLRP3 inflammasome is engaged in the process of cholestasis-induced liver injury and fibrogenesis (159). A growing body of data suggests that crosstalk occurs between various types of cell death (160). However, existing evidence explaining the involvement of NLRP3 in PSC remains extremely convoluted and contentious.

### 4.4 Inflammasomes and pyroptosis in IgG4-SC

Clinically, the progressive chronic liver illness known as IgG4-SC is quite similar to PSC. IgG4-SC is also a common complication of multifocal biliary strictures and IBD, and therefore, it may be misdiagnosed in some cases (161). In contrast to PSC, IgG4-SC is a multisystem fibroinflammatory disease that is defined by substantial lymphoplasmacytic infiltration of IgG4-positive cells and increased serum levels of IgG4 (162). Both the serum IgG4/IgG1 ratio and IgG4/IgG RNA ratio can be used to differentiate between the two conditions (163). Plasma ablator amplification makes it possible to recognize IgG4-SC with normal levels of IgG4. Compared with patients with PSC, those with IgG4-SC have a lower risk of cirrhosis, cholangiocarcinoma, and colorectal cancer. Steroids are currently the primary treatment option for IgG4-SC, but individuals with IgG4-SC who are resistant to steroids and immunosuppressants can benefit from rituximab (anti-CD20). Rituximab is a monoclonal antibody depletion therapy that targets the B-cell CD20 antigen (164).

#### 4.4.1 Differences in host–microbe interactions between IgG4-SC and PSC

IgG4-SC and PSC show both commonalities and differences in host–microbe interactions, which may be pertinent to the etiology of these illnesses and underscore the uniqueness of IgG4-SC (165). Intestinal secondary bile acids were decreased in both IgG4-SC and PSC mice. Reduced bacterial diversity and elevated numbers of potential pathogens, including *Veillonella*, *Enterococcus*, and *Clostridium*, have been observed in the gut microbiota of individuals with PSC (130, 166). There are potential links between microbial or metabolic features in PSC and cholestatic parameters, while disease-related genera and metabolites in IgG4-SC tend to be associated with transaminases of hepatocyte injury. According to the results of recent studies, liver inflammation of IgG4-SC may be responsible for the marked reduction in *Blautia* and elevation of succinate (167). However, further research is required to determine the function of inflammasomes in hepatocyte pyroptosis in IgG4-SC.

#### 4.4.2 Regulatory immune responses in IgG4-SC

A previous study found that the antigens IgG4-SC in patients with peripheral blood and tissue oligoclonal support the B-cell antigen-mediated reaction, and this environment is induced in genetically susceptible individuals with preexisting IgG4 switch B cells, which increase rapidly (168). Once regulatory immune responses are activated, memory Tregs are induced in the blood and infiltrate the affected tissue (169). Inflammation in IgG4-SC is stimulated by a mixture of cytokines that are derived from T cofactor 2, such as IL-4 and IL-13, or from Tregs, such as IL-10 and TGF-β (170).

TLRs and NLRs on monocytes and basophils, which are involved in innate immunity, are related to IgG4-SC lesions. The B-cell activator BAFF and IL-13 enhance IgG4 responses, which indicates that there is a crosstalk between the innate and adaptive immune systems (171). In addition, CCL23 and CCL25 chemokines, which are considered important biomarkers of the gut–liver axis (172, 173), are expressed in IgG4-SC-involved tissues. To fully understand the function of NLR family inflammasome-mediated hepatocyte pyroptosis in IgG4-SC, additional research is required.

## 5 Inflammasome-targeting therapies for AILDs

Numerous experimental studies on AILDs have demonstrated the function of the inflammasome in the fibrosis and liver injury caused by persistent inflammation (174). The results demonstrate that inhibiting the inflammasome and pyroptosis secretion might be effective therapeutic approaches of preventing inflammation-induced liver damage (175, 176). According to the latest data from preclinical experimental studies, we summarize the most up-to-date developments in inflammasome- and pyroptosis-targeted medicines for AILDs (**Table 1**).

**Table 1:** Potential Therapeutic Agents for autoimmune liver disease.

Targeting disease	Therapeutic agent	Targeting molecule	Species	Reference
AIH	Dimethyl fumarate	NLRP3 inhibitor	Mouse	(177, 178)
	Cucurbitacin E	NLRP3 inhibitor	Mouse	(179)
PBC	MCC950	NLRP3 inhibitor	Mouse	(158, 180–184)
	Paeoniflorin	NLRP3 inhibitor	Mouse	(185–187)
	Bongkrekic acid	APAF-1 inhibitor	Mouse; HepG2 cells	(131, 188)
	Schisandra phenol B	APAF-1 inhibitor; NLRP3 inhibitor	Mouse	(189–191)
PSC	IDN-7314	NLRP3 inhibitor	Mouse	(152, 192)
	Geniposidic acid	NLRP3 inhibitor	Mouse	(193, 194)

### 5.1 NLRP3 inhibitors

Dimethyl fumarate, a potential mitochondrial protective agent identified based on a screening of FDA-approved drug libraries, has been found to reduce serum inflammatory cytokine levels and relieve liver injury (177). Recent studies have shown that in addition to reducing mitochondrial damage and mtROS production, dimethyl fumarate can also regulate protein kinase A (PKA) signaling and inhibit NLRP3 inflammasome assembly, thereby alleviating ConA-induced liver injury in AIH (**Figure 2**) (178). The mechanism may involve the boosting of PKA signaling by dimethyl fumarate and increasing the phosphorylation of NLRP3 on Ser/Thr residues at PKA-specific sites to decrease the activation of the NLRP3 inflammasome. PKA pathway inhibitors (H89 and MLL-12330A) could counteract the protective effect of dimethyl fumarate on liver injury.

Cucurbitacin E glucoside has a significant hepatoprotective effect against ConA-induced AIH (179). The mechanism involves the upregulation of Sirtuin 1 (SIRT1), nuclear factor (erythroid-derived 2)-like 2 (Nrf2), and heme oxygenase 1 (HO-1) to reduce oxidative stress and the blocking of NF-κB/NLRP3 signaling to prevent NLRP3 inflammasome-mediated pyroptosis.

The diarylsulfonylurea compound MCC950, which is considered the most powerful and specific inhibitor of NLRP3 (180, 181), can reduce the fibrotic phenotype and reduce the expression of caspase-1 and IL-1β in the liver after chronic cholestatic liver injury (158). Additionally, MCC950 can switch macrophage differentiation to the M2 phenotype and reduce the liver injury severity in the PBC mouse model, which leads to an increase in the activity of immunosuppressive Tregs and results in the production of IL-10 and TGF-β (**Figure 3**) (182). By inhibiting autoreactive T cells, Tregs lead to the development of active tolerance to autoantigens and inhibit the occurrence of autoimmune diseases (183). At the same time, Tregs are a factor that hinders the removal of pathogens, which prolongs the course of chronic infection (184).

Paeoniflorin could reduce cholestatic inflammation and liver fibrosis in the PBC mouse model by eliminating mtROS through the SIRT1/forkhead box O1 (FOXO1)/superoxide dismutase 2 (SOD2) signaling pathway, and thereby alleviate mitochondrial damage and inhibit NLRP3 inflammasome activation (185–187).

Recent experimental studies have shown that a pan-cysteine protease inhibitor (IDN-7314) has the ability to stop NLRP3 inflammasome activation in *Mdr2* ^−/−^ mice (152), minimize liver injury, and reverse the serum bile acid profile as well as the features of the cholic acid-related microbiota (**Figure 4**) (192).

α-naphthalene isothiocyanate-induced cholestasis is known as the standard experimental model for PSC (193). Recent studies have found that the geniposidic acid in Oldenlandia diffusa Roxb can covalently bind to NLRP3 and inhibit the activation of NLRP3 inflammyome, thereby reducing α-naphthalene isothiocyanate induced cholestatic liver injury (194).

### 5.2 Apaf-1 inhibitors

Bongkrekic acid, which is a specific inhibitor of the mitochondrial endomembrane adenine nucleotide translocator (ANT), can strongly protect cells against bile acid-induced MPT and GSDME-dependent pyroptosis in a PBC mouse model and HepG2 cells (131). The underlying mechanism is that Bongkrekic acid can inhibit ANT-mediated MPT formation and APAF-1 assembly in BDL mice, thereby inhibiting APAF-1/caspase-11/GSDME-mediated pyroptosis (**Figure 3**). Such findings support the theory that ANT-mediated MPT may be responsible for bile acid-induced pyroptosis. ANT is a critical facilitator of the bile acid-induced MPT and the following APAF-1 assembly, which promotes rapid ATP release from mitochondria to the cytoplasm (188).

Schisandra phenol B is a bioactive substance isolated from *Schisandra chinensis*, which has protective effect against liver injury. Recent studies have found that schisandra phenol B could alleviate lithocholic acid-induced cholestatic liver injury through pregnane X receptor (PXR) (189). PXR can reportedly directly bind to FOXO1 and regulate its target to inhibit its activity (190), while FOXO1 can regulate the transcription of APAF-1, thereby inhibiting APAF-1/GSDME-mediated pyroptosis, considering the promoter of APAF-1 contains FOXO1 and FOXO3 binding sites. In addition, PXR can interact with NF-kB to inhibit NLRP3 inflammasome activation (191), thereby inhibiting NLRP3 inflammasome-mediated pyroptosis.

## 6 Discussion

Based on the latest experimental data, similarities and differences are observed in the proinflammatory mechanism and pyroptosis pathway among AIH, PBC and PSC. The proinflammatory pathway shared by the three diseases is the activation of NLRP3 inflammasome, which cleaves caspase-1 and promotes IL-1β secretion, thereby expanding the inflammatory response. In addition, PBC and PSC are mainly associated with gut microbiota microecology through aberrant immune system activation in the gut-liver axis. The gut–liver axis is a two-way communication pathway, and bile acids play an important role as mediators in the pathway (148). Through interhepatic circulation, bile acids can alter the quantity of the gut microbiota and impair intestinal barrier function through portal veins and bile ducts, leading to increased intestinal permeability (147, 195). As a result, MAMPs originating from the gut, such as lipopolysaccharides or microbial RNA, can enter the portal circulation and reach the liver, activating NLRP3 inflammasome and aggravating liver inflammation and fibrosis. Moreover, MAMPs and bile acids from the intestine in PBC can promote Kupffer cells to exhibit the M1 phenotype, secrete IL-6, TNF, and IL-1β, and aggravate liver injury and liver fibrosis. A comparison of the pyroptosis pathways of the three diseases showed that the typical pyroptosis pattern mediated by the NLRP3 inflammasome is predominant in AIH hepatocytes. However, two pyroptosis pathways are observed in PBC, and they are induced by bile acids, NLRP3/caspase-1/GSDMD, and APAF-1/caspase-11/GSDME (131). Interestingly, caspase-11 can be activated by bile acids, but it cannot cleave GSDMD to GSDMD-N, while caspase-11 can activate caspase-3 to cleave GSDME to induce pyroptosis. The mRNA level of caspase-11 increased in a PSC mouse model (152). Caspase-11 is a key molecule in pyroptosis; therefore, targeting caspase-11 may provide opportunities for new therapeutic interventions. However, no experimental study has verified typical and atypical pyroptosis in PSC.

IgG4-SC and PSC show both commonalities and differences in host–microbe interactions, which may be pertinent to the etiology of the illnesses and underscore the uniqueness of IgG4-SC (165). Intestinal secondary bile acids decreased in both IgG4-SC and PSC mice. Potential links are observed between microbial or metabolic features in PSC and cholestatic parameters, while disease-related genera and metabolites in IgG4-SC tend to be associated with transaminases of hepatocyte injury. However, further research is required to determine the function of inflammasomes in hepatocyte pyroptosis in IgG4-SC.

In addition, further experimental research on the inflammasome and pyroptosis in relation to intestinal microbiota and the gut–liver axis is required. As a potential key molecule in the gut–liver axis, NLRP6 is not only highly expressed in intestinal epithelial cells (45) but also specifically expressed in liver parenchymal cells (195). In alcoholic liver disease, the gut microbiota and its components, including LPS and dsDNA (196), activate the TLR4/MyD88–ROS pathway in the portal circulation, thereby triggering NLRP6 inflammasome-mediated pyroptosis (196). Intestinal microecology is known to be strongly correlated with the occurrence of AILDs (197). However, there have been no relevant experimental studies to verify the mechanism of the NLRP6 inflammasome in AILDs.

One prominent regulator of the innate immune response is the inflammasome (35). As a double-edged sword, NLRP3 may protect PSC during acute cholestatic liver injury by blocking other cell death pathways and aggravate liver injury and fibrosis during chronic cholestatic liver injury by inducing pyroptosis and promoting the release of inflammatory factors (158). In a PBC mouse model, moderate concentration of cholic acid (50 mM) activated caspase-9 to induce apoptosis, while high concentrations of bile acid (200 mM) activated caspase-11 induced pyroptosis (131). Overall, the available data suggest that inflammasome activation in AILDs primarily plays a proinflammatory and hepatoinjury-promoting role. However, the crosstalk between various types of cell death remains very complex and controversial (35).

At present, the drug treatments of AILDs mainly include immunoregulatory therapy, such as glucocorticoids and immunosuppressants. Immunosuppressive glucocorticoids, such as prednisone, are often used to reduce inflammation, but side effects can be severe. Steroids can weaken bones and cause vision problems, such as glaucoma (198). Azathioprine and 6-mercaptopurine reduce white blood cell counts and reduce resistance to infection. PBC and PSC are treated with ursodeoxycholic acid, the drug of choice for cholestatic liver disease (199, 200). However, based on the response criteria, this treatment fails in approximately 25% to 50% of patients (201). Therefore, a large proportion of patients still do not have adequate treatment, and understanding the mechanisms of the proinflammatory pathway in AILDs and potential new therapeutic approaches is critical. Experimental studies on AILDs have repeatedly demonstrated that the inflammasome is essential for the development of liver fibrosis and damage as a result of chronic inflammation (202). According to the latest available dta, dimethyl fumarate, cucurbitacin E, paeoniflorin, MCC950, IDN-7314, and geniposidic acid could inhibit the activation of NLRP3 inflammasome and the secretion of IL-1β, while bongkrekic acid and schisandra phenol B could inhibit the activation of APAF-1 and GSDME-dependent pyroptosis, thereby alleviating liver inflammatory damage in AILDs mouse models. However, few studies have focused on inflammasome-targeted therapy for AILDs. It is necessary to find and validate novel biological markers for inflammasome- and pyroptosis-associated signaling pathways and medicines for autoimmune liver illnesses through clinical trials and preclinical experimental investigations.

## Author contributions

JW andZS have contributed equally to this article. JW and ZS conceived and designed this article. JX, WJ and YC contributed to the revision of the chart. JW wrote the manuscript. GL and ZA jointly reviewed and revised the article. All authors have read and approved the submitted version of the manuscript.
